# Engineering properties of *Cassia tora* L. seeds and meal as a function of moisture content

**DOI:** 10.1038/s41598-022-12748-7

**Published:** 2022-05-23

**Authors:** Fei Peng, Fang Fang, Rui Xiang, Dan Liu

**Affiliations:** 1 School of Artificial Intelligence, Beijing Technology and Business University, Beijing, 100048 China; 2 College of Chemical Engineering, Zhengzhou University, Zhengzhou, 450001 China

**Keywords:** Engineering, Mathematics and computing

## Abstract

Engineering properties are of great importance for *Cassia tora* L. seeds in aspects of harvesting, handling mechanical design and product processing. The effect of moisture content (7, 10, 13, 16 and 19%) (wet basis) on the properties: physical (length, width, bulk and true density, porosity, thousand seeds mass, coefficient of static friction and angle of repose), mechanical (hardness, fragmentation energy and failure deformations), and thermal (specific heat, thermal conductivity and thermal diffusivity), were systematically studied. As the moisture contents increase from 7 to 19%, the length (*L*) increased from 4.52 to 5.87 mm, the thickness (*T*) from 2.51 to 3.21 mm and the width (*W*) from 2.36 to 3.02 mm, respectively. The bulk and true density of *Cassia tora* L. seeds decreased from 775.83 to 654.17 kg/m^3^ and from 1295.21 to 1154.72 kg/m^3^, respectively, with the moisture content raised from 7 to 19%. The thermal conductivity of *Cassia tora* L. seeds meal was found to be 0.068–0.098 W m^−1^ K^−1^, 0.078–0.112 W m^−1^ K^−1^, 0.089–0.125 W m^−1^ K^−1^, 0.098–0.136 W m^−1^ K^−1^, 0.108–0.148 W m^−1^ K^−1^, 0.119–0.159 W m^−1^ K^−1^, respectively, at 25 °C, 45 °C, 65 °C, 85 °C, 105 °C and 125 °C in moisture ranges of 7–19%. The thermal diffusivity was found to decrease from 5.21 × 10^–8^ to 4.53 × 10^–8^ m^2^/s, from 5.75 × 10^–8^ to 4.91 × 10^–8^ m^2^/s, from 6.11 × 10^–8^ to 5.17 × 10^–8^ m^2^/s, from 6.52 × 10^–8^ to 5.36 × 10^–8^ m^2^/s, from 7.17 × 10^–8^ to 5.77 × 10^–8^ m^2^/s, from 7.36 × 10^–8^ to 5.84 × 10^–8^ m^2^/s, respectively, at 25 °C, 45 °C, 65 °C, 85 °C, 105 °C and 125 °C in moisture ranges of 7–19%. The results suggested that physical properties exhibited linear relationships with moisture content using the regression model, while mechanical properties showed a second-order polynomial relationship with moisture content. Furthermore, significant variation existed in thermal properties because of differentiate moisture content and temperature. These data and rules are also useful for high efficiency machines design and mechanisms development.

## Introduction

*Cassia tora* L. is a small shrub widely grown in Asian countries^1,2^. It has tremendous medical values in the treatment of hyperlipidemia, hypercholesterolemia, hypertension, jaundice and etc^3^. In Chinese medical treatment, *Cassia tora* L. is used as indigenous medicine by mashing its original or toasted seeds, followed by boiling in water^4,5^. Besides, *Cassia tora* L. also functions as a healthy drink tea for improving visual acuity. Thus the release of the efficient substances, including chrysophenol, emodin, rhein and flavor, is highly dependent on the parameters of raw *Cassia tora* L. seeds material. Moreover, there is an increasing demand to attain the environment-depending physical, mechanical and thermal properties for equipment design in procedures from plantation to harvesting then transportation, storage, grinding and further processing of edible and medicinal filed. Mechanical properties of seeds are closely associated with the design of processing equipment such as grinding machines and other powder applications. Thermal properties are essential to understand their thermal behavior and to control heat transfer processes. Parameters concerning the physical, mechanical and thermal properties are especially beneficial to improve the working efficiency of the machine/operation and optimize the process parameters to improve the product quality.

The moisture content has been proved to exert great influences on physical, mechanical and thermal properties of many agricultural and sideline products, particularly crop seeds, for examples: sunflower^6^, quinoa^7^, arugulas^8^, yellow lentil^9^, fir species^10^ and so on. However, detailed measurements and analysis on the physical, mechanical and thermal properties due to various moisture contents have not been studied comprehensively for *Cassia tora* L. seeds.

Herein, this study focuses on influence of moisture content on properties: physical properties (including geometric dimensions, bulk and true density, porosity of accumulation, mass of 1000 seeds, angle of repose as well as coefficient of static friction against different sorts of contact material surfaces), mechanical properties (including hardness, fragmentation energy and failure deformations) of *Cassia tora* L. seed kernel, and thermal properties (including specific heat, thermal conductivity as well as thermal diffusivity) of *Cassia tora* L. seeds meal when their moisture content ranging from 7 to 19%.

## Materials and methods

### Sample preparation

Fresh *Cassia tora* L. seeds were purchased from the local farm produce market in Sichuan, China. The samples were cleaned to remove impurities like dirt, gravel and other foreign objects; meanwhile, damaged and immature seeds were also excluded. The original moisture content for the *Cassia tora* L. seeds was measured by putting the seeds into the drying oven and then kept the temperature at 105 ± 1 °C for 24 h^11^, which is found to be 6.13% wet basis (w.b). All samples were pre-treated in the oven to reach their initial moisture content before each test. Samples with moisture content of 7, 10, 13, 16 and 19% were prepared with 5 replications for each moisture content. It was carried out by sealing the pre-treated seeds with their initial moisture content and the calculated water in sealed polythene bags and then maintained them at temperature 5 °C for 15 days to guarantee the seeds with full penetration and adsorption of water. The water needed was calculated by the rewetting formula^12^:

1 $$Q = \frac{{W_{t} (M_{f} - M_{i} )}}{{(100 - M_{f} )}}$$

where *Q*—the weight of purified water that will be sprayed, *W*_t_—the initial weight of seeds sample, *M*_f_—the desired moisture content for seeds sample, and *M*_i_—the original moisture content for the seeds sample. Before testing, the seeds sample was allowed to equilibrate from 5 °C to room temperature in about 2 h’s time^13^.

### Measurements of physical properties

In order to attain the average size of *Cassia tora* L. seeds, the principal dimensions (namely thickness, length and width) of 100 randomly selected seeds were determined by vernier calipers with a precision of 0.01 mm.

Bulk density of *Cassia tora* L. seeds with various moisture contents was measured by adding seeds to a 1000 ml container, thus forming a 15 cm height volume by striking the top level. The bulk density refers to the weight of the sample divided by its volume. In addition, the averaged bulk density was calculated by repeating the above measurements ten times. The toluene (C_7_H_8_) displacement method was applied to measure true density of the *Cassia tora* L. seeds^7^. The porosity of *Cassia tora* L. seeds was determined and then calculated applying the bulk–true density relationship stated as follows^14^:

2 $$\varepsilon = \left( {1 - \frac{{\rho_{b} }}{{\rho_{t} }}} \right) \times 100$$

where *ε*—the porosity, *ρ*_*b*_—the bulk density, and *ρ*_*t*_—the true density.

Angle of repose refers to the geometric angle formed by the object and the horizontal plane when stacked. To be specific, *Cassia tora* L. seeds were placed in a topless and bottomless cylinder that has a diameter of 300 mm and a height of 500 mm, and then placed in the center of a flat circular plate. Then the cylinder was slowly raised, allowing the seeds to form a cone-like shape. Thus the angle of repose could be obtained by the ratio of the cone height to the cone diameter^11^.

The static friction coefficient of the sample on the four types of materials, specifically, plywood (µ_pl_), glass (µ_gl_), galvanized iron sheet (µ_ga_) and rubber (µ_ru_) was determined, respectively. It is depicted as follows: samples were placed on the surface of a platform with an adjustable tilt angle, and then gradually raised the tilt angle of the platform. When the sample starts to slide down, the ruler is used to record the tilt angle. The coefficient of friction can be calculated by the formula given as follows^8,15^:

3 $$\mu = \tan \alpha$$

where μ—the coefficient of friction, *α*—the tilt angle (°).

### Measurements of mechanical properties

The measurements of mechanical properties were carried out with slight modifications from the literature^16^. The hardness, fragmentation energy and failure deformations of *Cassia tora* L. seeds were measured by a texture analyzer (TMS-Pilot, FTC Company, United States) configured with a 75 mm-diameter compression probe. During tests, the operating conditions were set as following parameters: pre-test speed was set to 1.0 mm/s, test speed 0.5 mm/s, post-test speed 5.0 mm/s, and trigger force 0.20 N. Average value of 30 samples was collected for each moisture content.

### Measurements of thermal properties

In order to acquire the thermal properties of *Cassia tora* L. seeds, they were crushed by a small grinder (JFSD-100, Shanghai Jiading Grain and Oil Company, Ltd., China). The standard method of the 14-layer-sieve was utilized to measure the particle size distribution of *Cassia tora* L. seeds meal (ASAE S424.1). To be specific, the particle size distribution was determined by putting 100 g of the seed meal into an electric sieve shaker, which configured with fourteen sieve sizes for 10 min. Furthermore, the mean particle size value of the seed meal was obtained by the equation:

4 $$d_{gw} = \log^{ - 1} \left[ {\frac{{\sum {\left( {W_{i} \log \overline{d}_{i} } \right)} }}{{\sum {W_{i} } }}} \right]$$

5 $$\overline{{d_{i} }} = \left( {d_{i} \times d_{i + 1} } \right)^{\frac{1}{2}}$$

where *d*_*gw*_—the geometric mean diameter, $\overline{{d_{i} }}$– the geometric mean diameter of the sample on *i*th sieve, $d_{i}$—the sieve pore diameter of the *i*th sieve, $d_{i + 1}$—the sieve pore diameter in next larger than *i*th sieve, $W_{i}$—the sample mass on *i*th sieve.

The specific heat of *Cassia tora* L. seeds meal was measured by the differential scanning calorimetry (214 Polyma, Netzsch Company, Germany). It can be depicted as follows: first, adjust the instrument to the initial temperature (− 50 °C) by liquid nitrogen cooling. Second, the instrument equilibrates isothermally was applied and then dynamic scanning was performed with a heating rate of 10 °C/min form 25 °C to 120 °C. Then a “no-sample” run was carried out to provide the base-line, namely placed the weighed empty crucible in sample and reference holders and the measurement is performed within the set temperature range. Repeated the same operation for saphire standard as a reference and then for a 3–5 mg sample of *Cassia tora* L. seed meal. Each test is repeated three times, followed by calculating the average.

The thermal conductivity and thermal diffusivity were determined by Thermal Performance Analyzer (KD2 Pro, Decagon Company, United States). Each sample was tested at least three times and then the average of three times was obtained as the final result. All figures were generated using ORIGINPRO v2022 (https://www.originlab.com/origin).

## Results and discussions

### Physical properties

**Figure 1:**
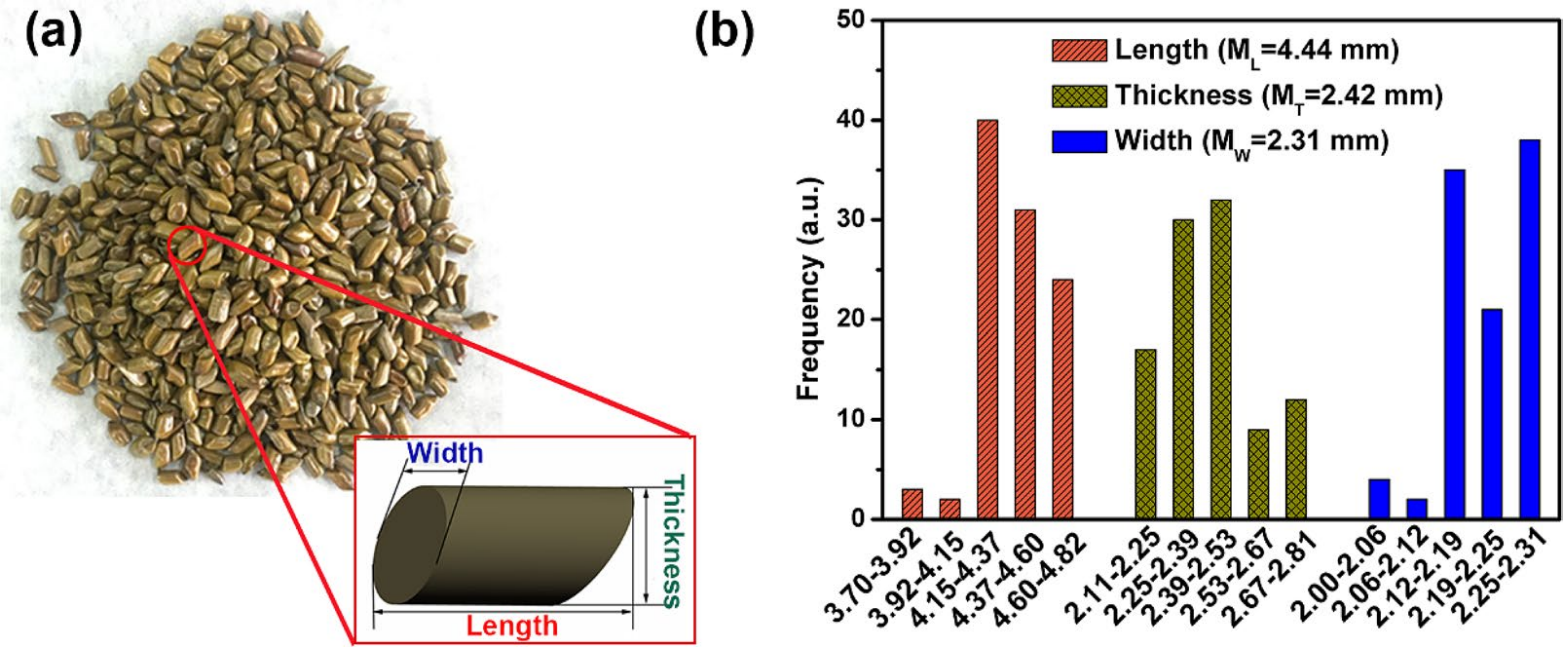
(**a**) Optical photo of untreated *Cassia tora* L. seeds and the magnified drawing of the seed with denoted dimensions (insert). (**b**) Histogram for the length, thickness and width of *Cassia tora* L. seeds at initial moisture content.

The typical optical image of *Cassia tora* L. seeds is given in Fig. 1a. It can be seen that it demonstrated a cuboid-like shape with oblique flanks. Length, thickness and width of the seeds were denoted as insert. Figure 1b shows that the pre-treated *Cassia tora* L. seeds with their initial moisture content have an average dimension of 4.44 mm × 2.42 mm × 2.31 mm (length × thickness × width) obtained from about 100 seeds.

**Figure 2:**
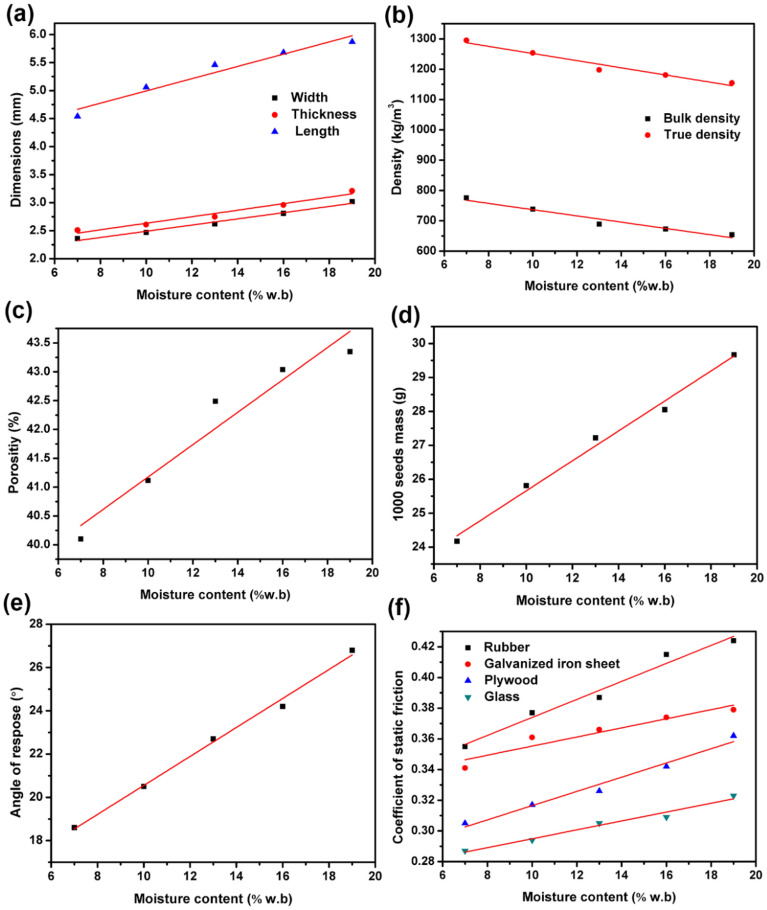
(**a**) Dimensions of *Cassia tora* L. seeds, (**b**) bulk and true densities, (**c**) porosity, (**d**) 1000 seeds mass, (**e**) angle of repose, (**f**) friction coefficient against four different material surfaces (rubber, galvanized iron sheet, plywood and glass, respectively) of *Cassia tora* L. seeds effected by moisture content of *Cassia tora* L. seeds.

The dimensions (length, thickness and width) of *Cassia tora* L. seeds determined with various moisture contents were exhibited, as shown in Fig. 2a. It can be seen that the dimensions of the *Cassia tora* L. seeds show a linear increase trend with the increased moisture contents. When moisture contents increase from 7 to 19% (w.b.), the length (*L*) increased from 4.52 to 5.87 mm, the thickness (*T*) from 2.51 to 3.21 mm and the width (*W*) from 2.36 to 3.02 mm, respectively. It can be linearly fitted with a regression equation of $L{ = }0.1093M{ + }3.9007$ with *R*^2^ = 0.9540, $T{ = }0.0583M + 2.0497$ with *R*^2^ = 0.9689, and $W{ = }0.0553M + 1.9367$ with *R*^2^ = 0.9851, respectively. Similar trends have been found for maize kernels^17^, millet^18^, lespedeza seeds^19^ and barley kernels^20^. This phenomenon is due to water imbibition of plant seeds^21^.

As depicted in Fig. 2b., the bulk and true density of *Cassia tora* L. seeds decreased from 775.83 to 654.17 kg/m^3^ and from 1295.21 to 1154.72 kg/m^3^, respectively, when moisture content varied from 7 to 19% (w.b.). It can also be concluded that the bulk density remains lower than the true density at identical moisture content. Figure 2b displays linear relationships between density and moisture content, thus linear regression equations of $\rho_{{\text{b}}} { = 11}{\text{.801}}x{ + 1369}{\text{.9}}$ with *R*^2^ = 0.9621 and $\rho_{{\text{t}}} { = }10.301M{ + 839}{\text{.86}}$ with *R*^2^ = 0.9537 were reached for bulk and true density, correspondingly.

Figure 2c shows that porosity increased from 40.10% to 43.35% when the moisture content varied from 7 to 19% (w.b.). Furthermore, they were observed to have a linearly relationship with a regression equation as $\varepsilon { = }0.2806M{ + 38}{\text{.37}}$ (*R*^2^ = 0.9420).

The 1000 seeds mass of *Cassia tora* L. seeds at different moisture contents is given in Fig. 2d. Mass of 1000 seeds of *Cassia tora* L. varies with moisture content and they show a positive linear relationship. The linear dependence of mass of 1000 seeds with moisture content could be represented by $W_{1000} { = }0.4413M{ + 21}{\text{.247}}$ with *R*^2^ = 0.9902.

The laboratorial angle of repose values raised from 18.6° to 26.8° when moisture content varied from 7 to 19% (Fig. 2e). The mathematical model for angle of repose and moisture content can be described by the formula depicted as: $\theta { = }0.67M{ + 13}{\text{.49}}$ with *R*^2^ = 0.9417. This finding agrees with the reports for rapeseed^22^, roasted bengal gram meal^23^ and soybean grains^24^. This may be due to the fact that adhesion of seed surface increased with increasing moisture content, hence it is more difficult to slip and roll. Therefore, angle of repose increased when moisture content increased within the studied range.

The coefficient of friction (*μ*) against four kinds of surfaces (rubber, galvanized iron sheet, glass and plywood) were shown in Fig. 2f. Analysis shows that the static coefficient of friction increase linearly with the increased moisture content and their relationship can be fitted as follows:

Fit. 1 $$\mu_{{{\text{ru}}}} { = }0.0059M{ + 0}{\text{.3153}}\quad \left( {R^{2} = 0.9768} \right)$$

Fit. 2 $$\mu_{{{\text{ga}}}} { = }0.0030M{ + 0}{\text{.3256}}\quad \left( {R^{2} = 0.9138} \right)$$

Fit. 3 $$\mu_{{{\text{pl}}}} { = }0.0046M{ + 0}{\text{.2702}}\quad \left( {R^{{2}} = 0.{9772}} \right)$$

Fit. 4 $$\mu_{{{\text{gl}}}} { = }0.0029M{ + 0}{\text{.2659}}\quad \left( {R^{{2}} = 0.{9764}} \right)$$

It is known that moisture on surface layer of biological material bound them together due to surface tension effect, and the adhesion force shows increasing tendency with the increased moisture content. Hence, it becomes relatively difficult for the seeds to roll and slide when they possess higher moisture content, resulting in the increase of static friction coefficient. These findings agreed with the report on seeds: dried ash gourd^25^, Jamun^26^, rapeseed^22^ and tamarind^27^.

### Mechanical properties

The hardness, fragmentation energy and failure deformations along thickness, width and length direction, namely *y*, *z* and *x* axis, respectively, were investigated at different moisture contents.

**Figure 3:**
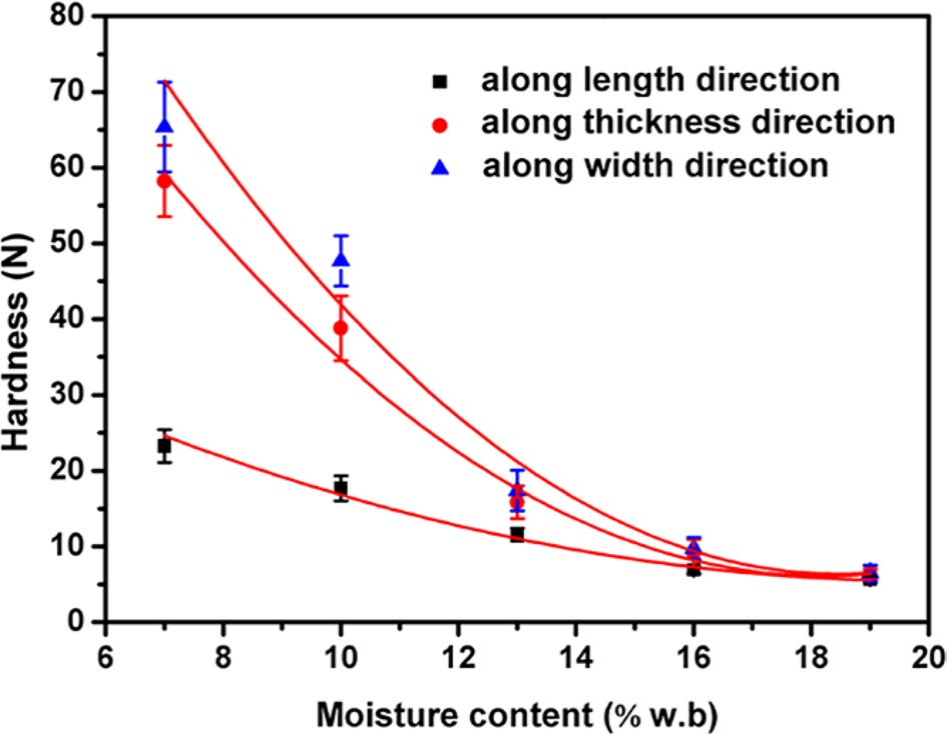
Relationship between moisture content and hardness of *Cassia tora* L. seeds measured along different directions.

The experimental values of the hardness at different loading directions were shown in Fig. 3. The highest hardness was observed along the width direction, followed by thickness direction and length direction. The hardness also varies with the wetness of the seed. The hardness decreases with increased moisture content along three directions. The mathematical model between the hardness and moisture content followed a second-order polynomial pattern, which can be written as follows:

Fit. 5 $${\text{X direction:}}\,H_{{\text{a}}} { = } 0.0802M^{2} - 3.6148M + 45.025\quad \left( {R^{{2}} = 0.{9931}} \right)$$

Fit. 6 $${\text{Y direction:}}\;H_{{\text{a}}} = 0.3929M^{2} - 14.6646M + 142.822\quad \left( {R^{{2}} = 0.{9795}} \right)$$

Fit. 7 $${\text{Z direction:}}\;H_{{\text{a}}} = 0.4087M^{2} - 15.8152M + 158.5286\quad \left( {R^{{2}} = 0.{9477}} \right)$$

These findings and conclusions are consistent with similar studies results obtained by wheat grain^12^ and olive Fruit^16^.

**Figure 4:**
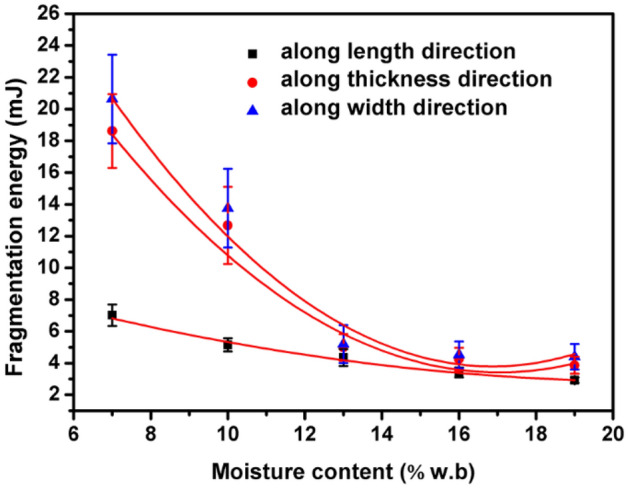
Relationship between moisture content and fragmentation of *Cassia tora* L. seeds measured along different directions.

Figure 4 shows the fragmentation energy along different directions of *Cassia tora* L. seeds affected by moisture content. The highest fragmentation energy was observed along width direction, followed by thickness direction and length direction. The experimental values of the fragmentation energy tend to decrease when moisture content increased from 7 to 19% along three directions, and there existed a quadratic curve correlation for fragmentation energy and moisture content of *Cassia tora* L. seeds. For the fragmentation energy, when the moisture content varies from 7 to 19% (w.b.), the corresponding regression equations were established as follows:

Fit. 8 $${\text{X direction:}}\;Fr{ = } 0.0209M^{2} - 0.8736M + 12.021\quad \left( {R^{{2}} = 0.{9918}} \right)$$

Fit. 9 $${\text{Y direction:}}\;Fr{ = } 0.1434M^{2} - \, 4.9925M + \, 46.971\quad \left( {R^{{2}} = 0.{9558}} \right)$$

Fit. 10 $${\text{Z direction:}}\;Fr{ = } 0.1697M^{2} - 0.58027M + 53.406\quad \left( {R^{{2}} = 0.{9566}} \right)$$

Besides, the hardness and fragmentation energy were highly correlated. Greater energy is needed to break the *Cassia tora* L. seeds of higher hardness^16,28,29^.

**Figure 5:**
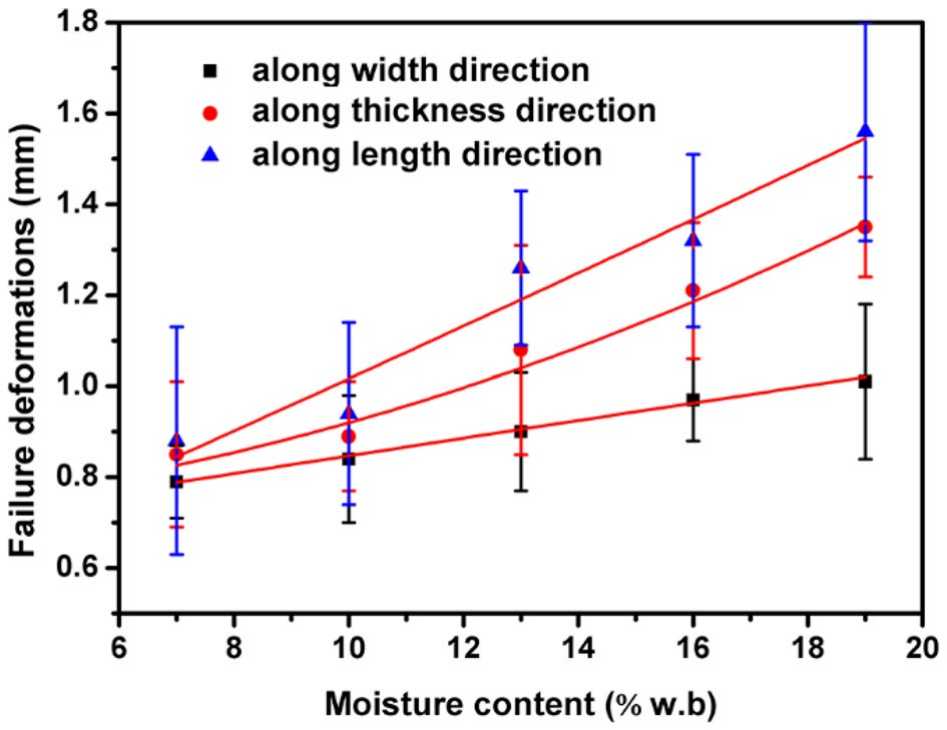
Relationship between moisture content and failure deformations of *Cassia tora* L. seeds measured along different directions.

The failure deformations with different moisture contents along different loading directions were presented in Fig. 5. The maximum failure deformations were observed along length direction, followed by width direction and thickness direction. The failure deformations increased quadratic linear when moisture contents increased. The corresponding regression equations were established for failure deformations of *Cassia tora* L. seeds with moisture content as follows:

Fit. 11 $${\text{X direction:}}\;F_{{\text{a}}} { = } 0.0008M^{2} { + }0.0176M + 0.6603\quad \left( {R^{{2}} = 0.{9971}} \right)$$

Fit. 12 $${\text{Y direction:}}\;F_{{\text{a}}} { = } 0.00146M^{2} + 0.00641M + 0.71026\quad \left( {R^{{2}} = 0.{9739}} \right)$$

Fit. 13 $${\text{Z direction:}}\;F_{{\text{a}}} { = } 0.000142M^{2} + 0.05468M + 0.45554\quad \left( {R^{{2}} = 0.{8697}} \right)$$

The results can be attributed to the internal structure bonding and hardness of the seeds. It is known that the tighter the internal structure bonding and the higher the hardness, the stronger the ability to endure load and resist cracking. When the moisture content increases, the internal structure of the seeds softens, so the ability to endure load and resist cracking load decreases, thus the hardness and fragmentation energy of *Cassia tora* L. seeds tends to decrease. As aforementioned, the dimensions of the seeds tended to increase with the increased moisture content, thus the failure deformations are proportional to the moisture content of *Cassia tora* L. seeds.

### Thermal properties

**Figure 6:**
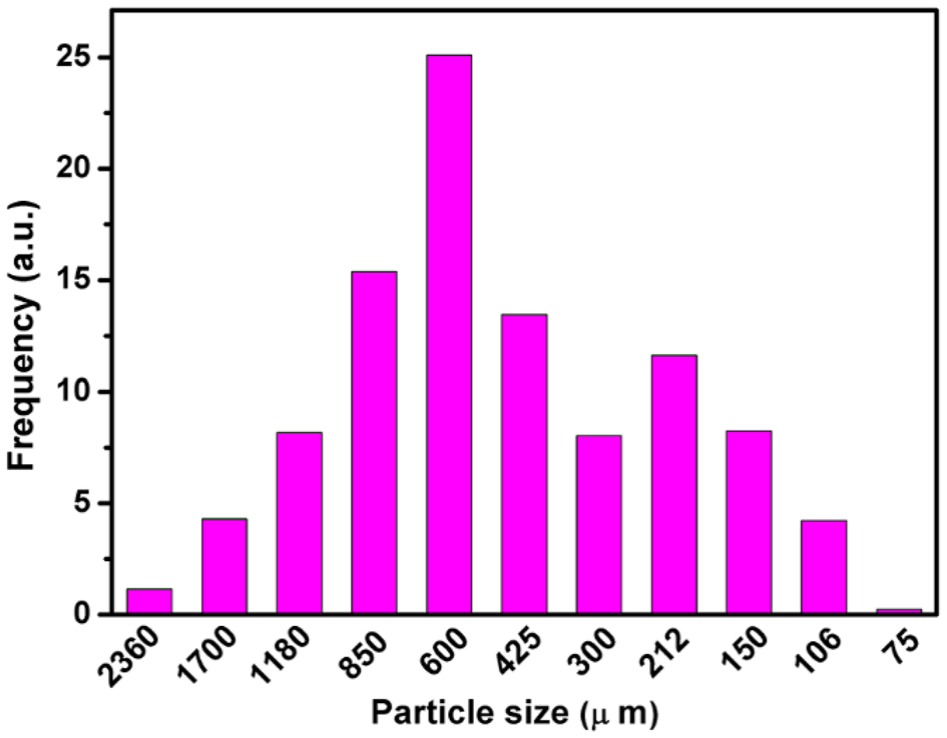
Histogram for particle size of the grinded *Cassia tora* L. seeds meal.

Thermal properties (specifically, specific heat, thermal conductivity and thermal diffusivity) are widely used in the engineering development and corresponding calculations related to thermal treatment of the seeds. The thermal-related properties were measured using the grinded seeds, which were notably influenced by particle size of the seeds meal^23^. Figure 6 shows the distribution of particle size of *Cassia tora* L. seeds meal was determined by the fourteen-layer sieve method^30^. The average particle size of the seeds meal used for the thermal measurement is thus determined to be 556.86 µm.

**Figure 7:**
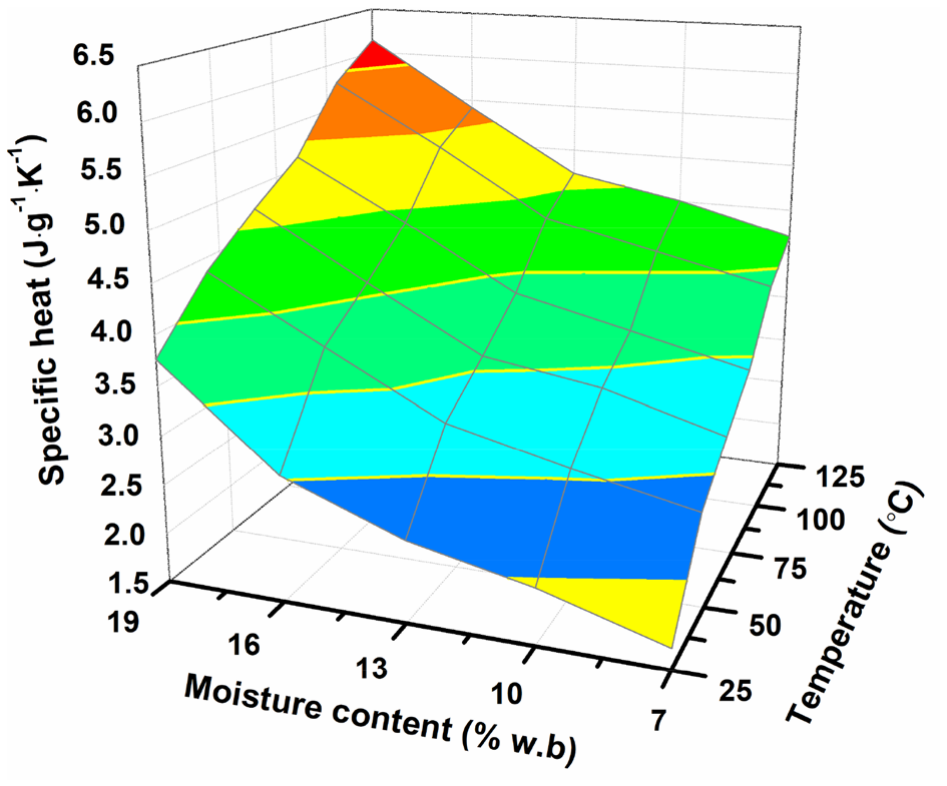
The specific heat (*C*_*p*_) of *Cassia tora* L. seeds meal affected by moisture content.

Since water has relatively specific heat and thermal conductivity, temperature and moisture content exert a great influence on the thermal properties of seeds. Therefore, a slight increment of moisture content results in significant increment in specific heat value (Fig. 7.). When the moisture content varied from 7 to 19%, the specific heat enlarged from 1.714 to 3.764 J·g^−1^ K^−1^, 2.568 to 4.354 J g^−1^ K^−1^, 2.935 to 4.769 J g^−1^ K^−1^, 3.282 to 5.122 J·g^−1^ K^−1^, 3.892 to 5.777 J·g^−1^ K^−1^ and 4.197 to 6.141 J·g^−1^ K^−1^ at 25 °C, 45 °C, 65 °C, 85 °C, 105 °C and 125 °C. Similar reports were also found in gelatin-free marshmallow^31^, green bean seed^32^ and lespedeza seeds^19^.

Fit. 14 $$C_{P} { = } 0.{98 + }6.{62} \times {1}0^{ - 2} M + {2}{\text{.32}} \times {1}0^{ - 2} T\quad \left( {R^{{2}} = 0.{98}0{7}} \right)$$

**Figure 8:**
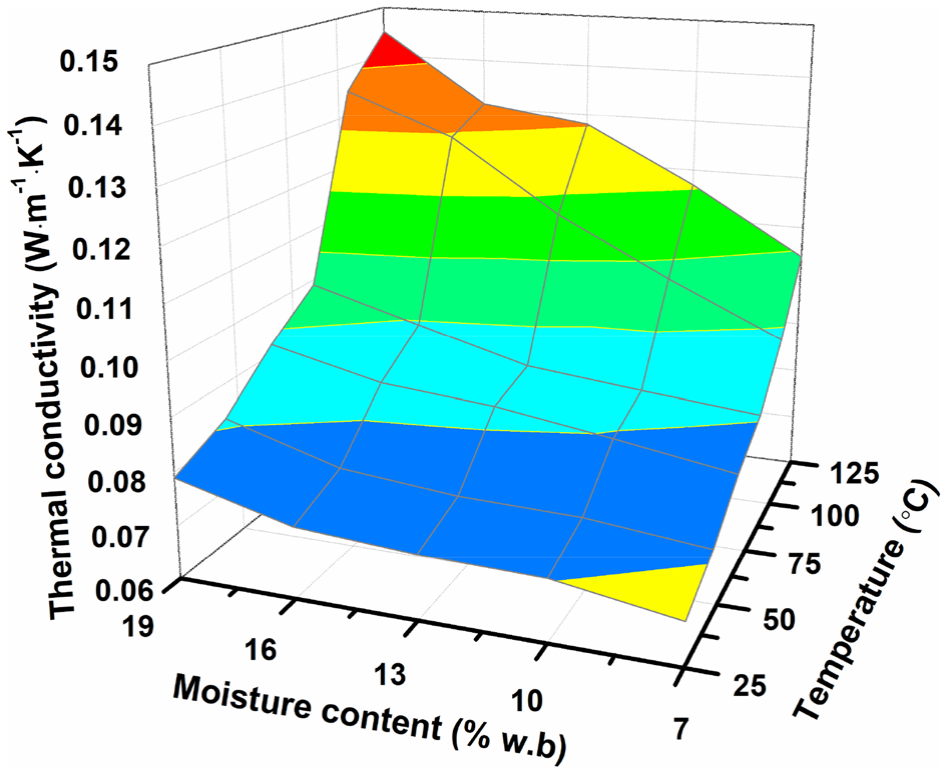
Thermal conductivity (*k*) of grinded *Cassia tora* L. seeds meal affected by moisture content.

The thermal conductivity of *Cassia tora* L. seeds meal, as shown in Fig. 8, was discovered to be 0.068–0.098 W·m^−1^ K^−1^, 0.078–0.112 W·m^−1^ K^−1^, 0.089–0.125 W m^−1^ K^−1^, 0.098–0.136 W m^−1^ K^−1^, 0.108–0.148 W·m^−1^ K^−1^, 0.119–0.159 W·m^−1^ K^−1^, respectively, at 25 °C, 45 °C, 65 °C, 85 °C, 105 °C and 125 °C in moisture ranges of 7–19%. Thermal conductivity enlarged with increased moisture content and temperature. Similar phenomena and laws of thermal conductivity have also been discovered related to pomegranate (*Punica granatum*) fruit^33^, sesame seed gum^34^, soybean seeds^24^, and watermelon seeds^35^. Thermal conductivity can quadratic functioned with moisture content (*M*) and temperature (*T*) using multiple regression analysis:

Fit. 15 $$k = 0.046 + 0.01M + 6.61 \times 10^{ - 5} M^{2} - 2.14 \times 10^{ - 7} T^{2} + 1 \times 10^{ - 5} MC\quad \left( {R^{2} = 0.984} \right)$$

**Figure 9:**
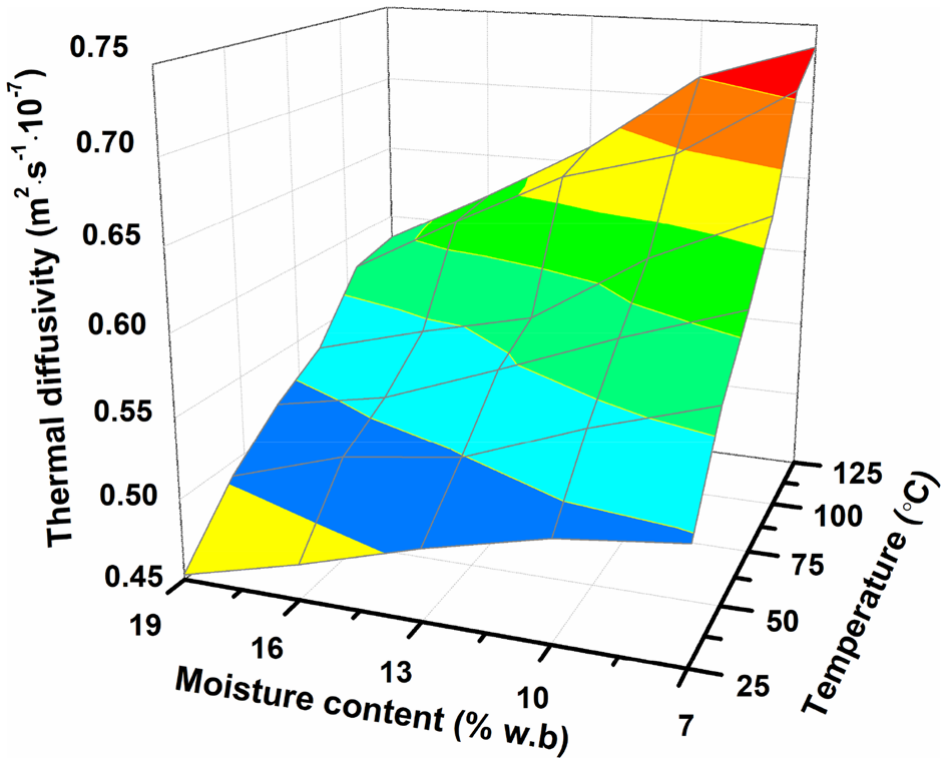
Thermal diffusivity (*α*) of grinded *Cassia tora* L. seeds affected by moisture content.

The curves and change laws between moisture contents and thermal diffusivity of seed meal at various temperatures are shown in Fig. 9. The results show that the thermal diffusivity increased as moisture contents increased. The thermal diffusivity was found to decrease from 5.21 × 10^–8^ to 4.53 × 10^–8^ m^2^/s, from 5.75 × 10^–8^ to 4.91 × 10^–8^ m^2^/s, from 6.11 × 10^–8^ to 5.17 × 10^–8^ m^2^/s, from 6.52 × 10^–8^ to 5.36 × 10^–8^ m^2^/s, from 7.17 × 10^–8^ to 5.77 × 10^–8^ m^2^/s, from 7.36 × 10^–8^ to 5.84 × 10^–8^ m^2^/s, respectively, at 25 °C, 45 °C, 65 °C, 85 °C, 105 °C and 125 °C in moisture ranges of 7–19%. An increasing trend in thermal conductivity of the grinded *Cassia tora* L. seeds was also observed with the increase in temperature. Regression analysis in the fitting equation showed that there is also a multiple relationship for thermal diffusivity (*α*), moisture content (*M*) together with temperature (*T*) as follows:

Fit. 16 $$\alpha \times 10^{ - 7} = 0.5 - 0.5 \times 10^{ - 2} M + 4.49 \times 10^{ - 5} M^{2} + 0.03 \times T - 4.20 \times 10^{ - 7} T^{2} - 7.34 \times 10^{ - 5} MC\quad \left( {R^{2} = 0.986} \right)$$

The facts that thermal diffusivity of Cassia tora seed meal showed a decreasing trend with increased moisture content and increase with increased temperature, are identical with some previous findings, such as for seeds: watermelon seeds^36^, jack bean seeds^37^, and green bean seed^32^.

## Conclusions

Systematic and comprehensive research on physical, mechanical and thermal properties of *Cassia tora* L. seeds and meal was carried out, and significant variation in these properties was analyzed due to the level of moisture content. Specifically, The findings obtained revealed that diameter, thickness, thickness, porosity, 1000 seeds mass, angle of repose, and coefficient of friction enlarged linearly as moisture content increased. However, bulk and true density both reduced linearly as moisture content increased. Within the range of moisture contents considered, rubber material possessed the highest friction coefficient, then followed by galvanized iron sheet, plywood and glass. Hardness and fragmentation energy decreased with increased moisture content, while failure deformations increased with increased moisture content. In addition, there is a quadratic function relationship between hardness, fragmentation and failure deformations energy with moisture content, respectively. The specific heat and thermal conductivity are positively correlated to moisture content, respectively, but thermal diffusivity has an inverse relationship.

In this article, physical, mechanical and thermal parameters of the *Cassia tora* L. seeds were formulated in regression equations with changing factors. Since moisture content is changeable during the procedure of storage and operation processes, these equations and laws are extremely helpful to obtain the physical, mechanical and thermal parameters of *Cassia tora* L. seeds under various conditions. These data and rules have important guidance significance for high efficiency machines design, mechanisms development and processing parameters optimization related to *Cassia tora* L. seeds and meal.

## Data Availability

The data presented in this study are available on request from the corresponding author (FP).
